# HPV E6/E7 promotes aerobic glycolysis in cervical cancer by regulating IGF2BP2 to stabilize m^6^A-MYC expression

**DOI:** 10.7150/ijbs.67770

**Published:** 2022-01-01

**Authors:** Chenchen Hu, Tianyue Liu, Chenying Han, Yuxin Xuan, Dongbo Jiang, Yuanjie Sun, Xiyang Zhang, Wenxin Zhang, Yiming Xu, Yang Liu, Jingyu Pan, Jing Wang, Jiangjiang Fan, Yinggang Che, Yinan Huang, Jiaxing Zhang, Jiaqi Ding, Shuya Yang, Kun Yang

**Affiliations:** 1 Department of Immunology, Air Force Medical University (The Fourth Military Medical University), Xi'an, Shaanxi, 710032, China.; 2 School of Basic Medicine, Air Force Medical University (The Fourth Military Medical University), Xi'an, Shaanxi, 710032, China.

**Keywords:** HPV E6/E7, IGF2BP2, cervical cancer, aerobic glycolysis, m^6^A-MYC

## Abstract

Enhanced aerobic glycolysis constitutes an additional source of energy for tumor proliferation and metastasis. Human papillomavirus (HPV) infection is the main cause of cervical cancer (CC); however, the associated molecular mechanisms remain poorly defined, as does the relationship between CC and aerobic glycolysis. To investigate whether HPV 16/18 E6/E7 can enhance aerobic glycolysis in CC, E6/E7 expression was knocked down in SiHa and HeLa cells using small interfering RNA (siRNA). Then, glucose uptake, lactate production, ATP levels, reactive oxygen species (ROS) content, extracellular acidification rate (ECAR) and oxygen consumption rate (OCR) were evaluated. RNA-seq was used to probe the molecular mechanism involved in E6/E7-driven aerobic glycolysis, and identified IGF2BP2 as a target of E6/E7. The regulatory effect of IGF2BP2 was confirmed by qRT-PCR, western blot, and RIP assay. The biological roles and mechanisms underlying how HPV E6/E7 and IGF2BP2 promote CC progression were confirmed *in vitro* and *in vivo*. Human CC tissue microarrays were used to analyze IGF2BP2 expression in CC. The knockdown of E6/E7 and IGF2BP2 attenuated the aerobic glycolytic capacity and growth of CC cells, while IGF2BP2 overexpression rescued this effect *in vitro* and *in vivo*. IGF2BP2 expression was higher in CC tissues than in adjacent tissues and was positively correlated with tumor stage. Mechanistically, E6/E7 proteins promoted aerobic glycolysis, proliferation, and metastasis in CC cells by regulating MYC mRNA m^6^A modifications through IGF2BP2. We found that E6/E7 promote CC by regulating MYC methylation sites via activating IGF2BP2 and established a link between E6/E7 and the promotion of aerobic glycolysis and CC progression. Blocking the HPV E6/E7-related metabolic pathway represents a potential strategy for the treatment of CC.

## Introduction

Cervical cancer (CC) is the most common cancer among women in 28 countries and the fourth leading cause of cancer-related death in women globally 1. In the United States, an estimated 13,800 cases of invasive CC were diagnosed in 2020, resulting in 4290 deaths 2. Despite increasing efforts, CC-related mortality remains high 3, mainly due to its unclear pathogenesis. Identifying novel therapeutic targets is expected to further the understanding of the pathogenesis of CC and expand the scope of treatment for this disease.

Human papillomavirus (HPV; family Papillomaviridae) is a small, non-enveloped virus containing a circular double-stranded DNA genome. HPV mainly infects basal keratinocytes of poorly differentiated squamous epithelia and drives the tumorigenesis of CC, head and neck cancer, and oropharyngeal cancer, among others. Persistent infection with high-risk HPV is considered to be the main cause of CC 4, with the most common genotypes being human papillomavirus type 16 and 18 (HPV16/18) 2. HPV infection alters the metabolism of tumor cells, resulting in immunosuppression and immune evasion, and, consequently, the promotion of tumorigenesis 5. Changes in the metabolic phenotype are one of the markers of the post-infection microenvironment 6. The post-HPV infection microenvironment actively instructs the metabolic activity of tumor cells, a key factor in HPV transmission and malignant CC progression 7.

Metabolic reprogramming due to oncogenic mutations is increasingly recognized as playing an important role in the tumorigenesis of several cancers, including CC 8. The activity of glucose transporters and key enzymes in aerobic glycolysis is enhanced to support the rising glucose demand of cells in the tumor microenvironment (TME), thereby promoting their proliferation 9. Cancer cells can also regulate mitochondrial metabolic pathways in a TME-dependent manner, which increases their resistance to apoptosis and promotes their survival 10. Although the therapeutic targeting of glucose metabolism-related pathways and molecules can change tumor immunity and attenuate the growth of tumor cells, the associated molecular mechanisms are poorly understood.

RNA binding proteins (RBPs) are key effectors of gene expression, regulating RNA metabolism at multiple levels, and form a large regulatory network involved in maintaining cell homeostasis 11. RBP dysregulation is associated with many diseases, including cancer 12. RBPs can promote cancer cell metastasis by stabilizing mRNA, thereby increasing protein expression 13. Additionally, RBPs can target and modulate the activity of metabolism-associated molecules 12. Insulin-like growth factor-2 mRNA binding proteins (IGF2BPs), including IGF2BP1, IGF2BP2, and IGF2BP3, can promote tumor progression by stabilizing methylated mRNA as well as enhancing tumor cell proliferation 14, 15.

To date, the function of IGF2BP2 in CC has not been systematically investigated. Here, we found that the knockdown of E6/E7 in CC cells significantly attenuated their proliferative and metastatic abilities as well as their capacity for aerobic glycolysis *in vitro* and *in vivo*. RNA-sequencing (RNA-seq) results showed that IGF2BP2 expression was significantly downregulated in E6/E7-knockout cells. Moreover, the expression of IGF2BP2 was significantly upregulated in tumor tissues compared with that in adjacent tissues and was positively correlated with CC stage. Mechanistically, we found that E6/E7 proteins regulate the activity of key enzymes in the aerobic glycolysis pathway by affecting the binding of IGF2BP2 to MYC m^6^A sites. We further established that the overexpression of IGF2BP2 in E6/E7-knockout CC cells rescued glycolytic flux. Combined, our results indicate that IGF2BP2 is a downstream target of E6/E7 and plays a role in the progression of CC.

## Materials and methods

### Cell culture

The human CC cell lines SiHa and HeLa were cultured in Dulbecco's modified Eagle's medium (DMEM) (Gibco, USA) supplemented with 10% FBS (Gibco) at 37 °C under 5% CO_2_. Both cell lines tested mycoplasma-negative and were authenticated using short tandem repeat (STR) profiling 16.

### RNA interference and generation of stable cell lines

Small interfering RNAs (siRNAs) targeting HPV16 E6/E7, HPV18 E6/E7, IGF2BP2, and methyltransferase-like 14 (METTL14) were purchased from GenePharma (Shanghai, China). siRNA and negative control (NC) siRNA transfection was performed with Lipofectamine 3000 reagent (Invitrogen, USA) for 48 h. The transfection efficiency was verified by qRT-PCR and western blotting.

The HPV16 E6/E7-shRNA lentivirus, HPV18 E6/E7-shRNA lentivirus, and NC-shRNA were constructed by GenePharma (Shanghai, China). LV17-NC and LV17-Homo IGF2BP2 were cloned into the pcDNA3.1 (+) vector. The CC cells were infected with lentiviruses and cultured in DMEM supplemented with 5 µg/ml polybrene. To rescue the effect of E6/E7 knockdown on CC cells, HPV16/18 E6/E7-knockdown cells were infected with the IGF2BP2-overexpression lentivirus. Stable cell lines were generated by selection with 10 µg/ml puromycin (Solarbio, China). The expression of HPV16 E6/E7, HPV18 E6/E7, and IGF2BP2 was validated by qRT-PCR and western blot. The siRNA, shRNA and plasmid sequences are listed in Table S1.

### Quantitative real-time reverse transcription-quantitative PCR (qRT-PCR) and RNA-seq analysis

Total RNA was isolated from treated CC cells using an RNA extraction kit and reverse transcribed into cDNA by one-step RT-PCR. Real-time qPCR was performed using SYBR Green PCR Master Mix (TaKaRa, Tokyo, Japan). The sequences of the primers used are shown in Table S2.

Differentially expressed genes in HPV16 E6/E7-knockout SiHa cells were identified by RNA-seq analysis which was performed by Lc-Bio Technologies (Hangzhou) Co., Ltd (China).

### Western blot and immunoprecipitation (IP) assays

Protein expression was measured by western blot as previously described 17. After blocking, the polyvinylidene difluoride membranes were incubated with different antibodies (Table S3) overnight at 4 °C.

For co-IP, transfected SiHa cells were lysed using lysis buffer containing a protease and phosphatase inhibitor cocktail. After centrifugation of the lysed cells at 4 °C for 10 min, the supernatant was collected. Anti-m^6^A and IgG antibodies were added to the lysate with Protein A/G PLUS-Agarose, followed by overnight incubation. Finally, the beads were washed with IP buffer and analyzed by western blot.

### Cell viability and colony formation assays

Cell viability was monitored using a Cell Counting Kit-8 (CCK-8) assay (Yisheng, China). Cells transfected with siRNAs and their corresponding NCs were seeded into 96-well plates (5000 cells/well). After incubation with DMEM containing 10% CCK-8 reagent for 2 h, the absorbance was measured at 450 nm.

For the colony formation assay, transfected cells were seeded in 60-mm dishes (400 cells/dish). After 2 weeks, the resulting cell colonies were fixed in 4% paraformaldehyde (PFA) and stained with 1% crystal violet (Solarbio) for 15 min. After washing with PBS, the number of colonies was counted using ImageJ software.

### 5-Ethynyl-2′-deoxyuridine (EdU) incorporation assay

EdU assays were performed using a BeyoClick EdU Cell Proliferation Kit with DAB (Beyotime, China). After incubation with 10 µM EdU for 2 h, the cells were fixed in 4% PFA for 15 min, permeabilized in PBS containing 0.3% Triton X-100, and stained using Click Additive Solution at 37 °C for 1 h. Nuclei were counterstained with DAPI (Beyotime). Images were captured using an EVOS M5000 Fluorescence Microscope (Thermo Fisher Scientific, USA).

### Cell migration and transwell assays

The real-time cell analysis (RTCA) system was used to continuously monitor cell migration for 30 h. Migration was measured using a 16-well CIM plate, which was divided into upper and lower chambers by 8-µm microporous membranes. The migration curves were recorded by RTCA software.

The transwell invasion assay was performed in transwell chambers (Thermo Fisher Scientific) precoated with Matrigel. Transfected SiHa and HeLa cells were treated with mitomycin C, suspended in serum-free medium, and seeded into the upper chamber. DMEM supplemented with 10% FBS was placed into the lower chamber as a chemoattractant. After incubation for 24 h, the culture medium was discarded, and the cells were fixed, stained, and photographed.

### TUNEL assay

The TUNEL assay 18 was performed on mouse tumor sections using a TUNEL BrightGreen Apoptosis Detection Kit (Vazyme, China). Slides containing mouse tumor tissue were dewaxed, rehydrated, permeabilized with proteinase K, and incubated with the recombinant TdT enzyme mixture at 37 °C for 1 h. After washing with PBS, the slides were stained with 2 µg/ml DAPI for 5 min in the dark.

### Chromatin immunoprecipitation (ChIP)-qPCR

The ChIP assay 19 was performed using a SimpleChIP Plus Enzymatic Chromatin IP Kit (Magnetic Beads) (Cell Signaling Technology, USA) according to the manufacturer's protocols. Specifically, approximately 1.2×10^7^ SiHa cells were cross-linked in 1% formaldehyde; after 10 min, glycine was added to terminate the reaction. Adherent cells were scraped off in PBS containing a protease inhibitor cocktail and centrifuged at 2000 × g for 5 min to collect the precipitate. Micrococcal nuclease was added to digest the DNA to the optimal length and 0.5 M EDTA was used to stop the digest. The nuclear membrane was then disrupted by ultrasound and the supernatant containing the cross-linked chromatin sample was collected. Rabbit monoclonal anti-MYC (Cell Signaling Technology) antibody, IgG antibody, and samples were incubated overnight at 4 °C for subsequent immunoprecipitation. ChIP-grade protein G magnetic beads were added to each IP reaction followed by shaking for 2 h. Then, the beads were cleaned with high/low salt solutions, the chromatin was eluted, and cross-linking was reversed. DNA was purified using spin columns and subjected to qRT-PCR. The primers used for ChIP-qPCR are listed in Table S2.

### RNA immunoprecipitation

RNA immunoprecipitation (RIP) 19 was performed using a RIP-Assay Kit (MBL Life Science, Japan). After transfection in a 15-cm dish for 48 h, adherent SiHa cells were collected using cell scratchers and suspended in nuclease-free PBS. For co-immunoprecipitation, 50% bead slurry and 15 µg of antibody (anti-IGF2BP2 for target RNA binding protein and IgG as a negative control) were added to new nuclease-free tubes. The precleared cell lysate was transferred to the tube containing Antibody-immobilized Protein A/G PLUS-Agarose beads, washed once with lysis buffer, and incubated with rotation for 3 h at 4 °C. The antibody-immobilized beads-RNP complex was obtained by centrifugation. The complex was washed three times with wash buffer and RNA was isolated for subsequent qRT-PCR analysis.

### m^6^A-RNA immunoprecipitation (Me-RIP)

For Me-RIP, an N6-methyladenosine (m^6^A) antibody (Active Motif, China) was used to pull down m^6^A-modified MYC. Total RNA was extracted from treated SiHa cells and purified using a polyA Spin mRNA Isolation Kit (NEB, China). The IP buffer was prepared as previously described 20. Antibody-immobilized Protein A/G PLUS-Agarose beads were prepared by incubating with m^6^A antibody and IgG antibody in IP buffer. The purified RNA was then added to the above-mentioned microcentrifuge tubes containing protease and RNase inhibitors followed by incubation at 4 °C overnight. The RNA was extracted using an RNA extraction kit and analyzed by qRT-PCR, the primers used for qPCR are listed in Table S2. The protein expression of m^6^A was detected by IP assay. Briefly, 100 µl of the mixture containing the antibody-immobilized beads-RNP complex was added to a new tube, resolved by SDS-PAGE, and subjected to western blotting.

### Immunohistochemistry (IHC)

The CC tissue microarrays (TMAs) FDU961 and FDU1921 (Taibsbio, China) were stained with antibodies targeting IGF2BP2 (1:50, Proteintech), MYC (1:100, Proteintech), and Ki-67 (1:300; Abcam) as previously described 21. Stained sections were scanned using a 3DHISTECH imaging system (Hungary) and analyzed by Indica Labs software (USA). A Servicebio image analysis system was used to automatically read the measurement area of the tissue sections and calculate the histochemistry score (H-score) in the measurement area.

### Flow cytometry

Flow cytometry was used to detect the effect of HPV16/18 E6/E7 and IGF2BP2 on cell apoptosis and the cell cycle in HeLa and SiHa cells transfected with siRNAs or plasmids. Cell apoptosis was assessed using an Annexin V-FITC/PI apoptosis double staining kit while the cell cycle was analyzed using PI/RNase staining buffer (BD, USA) according to the manufacturers' instructions. NovoExpress software (ACEA Biosciences, USA) was used for data acquisition and analysis.

### Metabolic assay

Glucose uptake and lactate production in CC cell-culture medium were assayed using commercial kits (Biovision, Milpitas, CA, USA) and ATP production was measured using an ATP Assay Kit (Beyotime) following the manufacturers' protocols 22. Intracellular reactive oxygen species (ROS) levels were assessed using the fluorescent dye 2′-7′-dichlorofluorescin diacetate (DCFH-DA) (Beyotime) and analyzed by flow cytometry 23.

### Extracellular acidification rate (ECAR) and oxygen consumption rate (OCR)

Metabolic indicators (ECAR, OCR) were used to evaluate glycolytic fluxes. Treated SiHa cells were evenly spread on a 24-well XF cell culture plate (Seahorse Bioscience, USA) at a density of 5×10^4^ cells per well. After allowing the cells to adhere overnight, the culture medium was discarded and fresh assay medium containing different detection reagents was added into each well. The cartridge was loaded with glucose (10 mM), oligomycin (1 μM), and 2-deoxyglucose (2-DG, 50 mM) to detect ECAR at specified time points. Drug concentrations for OCR detection were as previously described 24, 25.

### Animal experiments

Female BALB/c nude mice (4-6 weeks old) were randomly allocated to four groups (*n*=7 per group) to establish a xenograft tumorigenesis model and observe survival time. SiHa cells stably expressing shNC, shIGF2BP2, shHPV16E6/E7+oe-NC, or shHPV16E6/E7+oe-IGF2BP2 were subcutaneously injected into nude mice (5×10^6^ cells per mouse). The tumor volume was measured every two days after tumor formation. Tumor volume was calculated as V=1/2×length×width^2^. Images were acquired using a PerkinElmer IVIS preclinical *in vivo* imaging system. Survival data were also recorded for each mouse. The animal study was reviewed and approved by the Ethics Committee of the Fourth Military Medical University.

### Statistical analysis

GraphPad Prism 8.0.2 was used for statistical analysis and graphing. The Student's *t*-test was used for comparisons between two groups and one-way ANOVA was used for comparisons among multiple groups. The survival probability was calculated using the Kaplan-Meier method. Each experiment was repeated at least three times and the data are presented as means ± SD. *p < 0.05, **p < 0.01, ***p < 0.001, ****p < 0.0001. NS, not significant.

## Results

### The downregulation of HPV16/18 E6/E7 suppresses proliferation, metastasis, and aerobic glycolysis in CC cell lines

**Figure 1:**
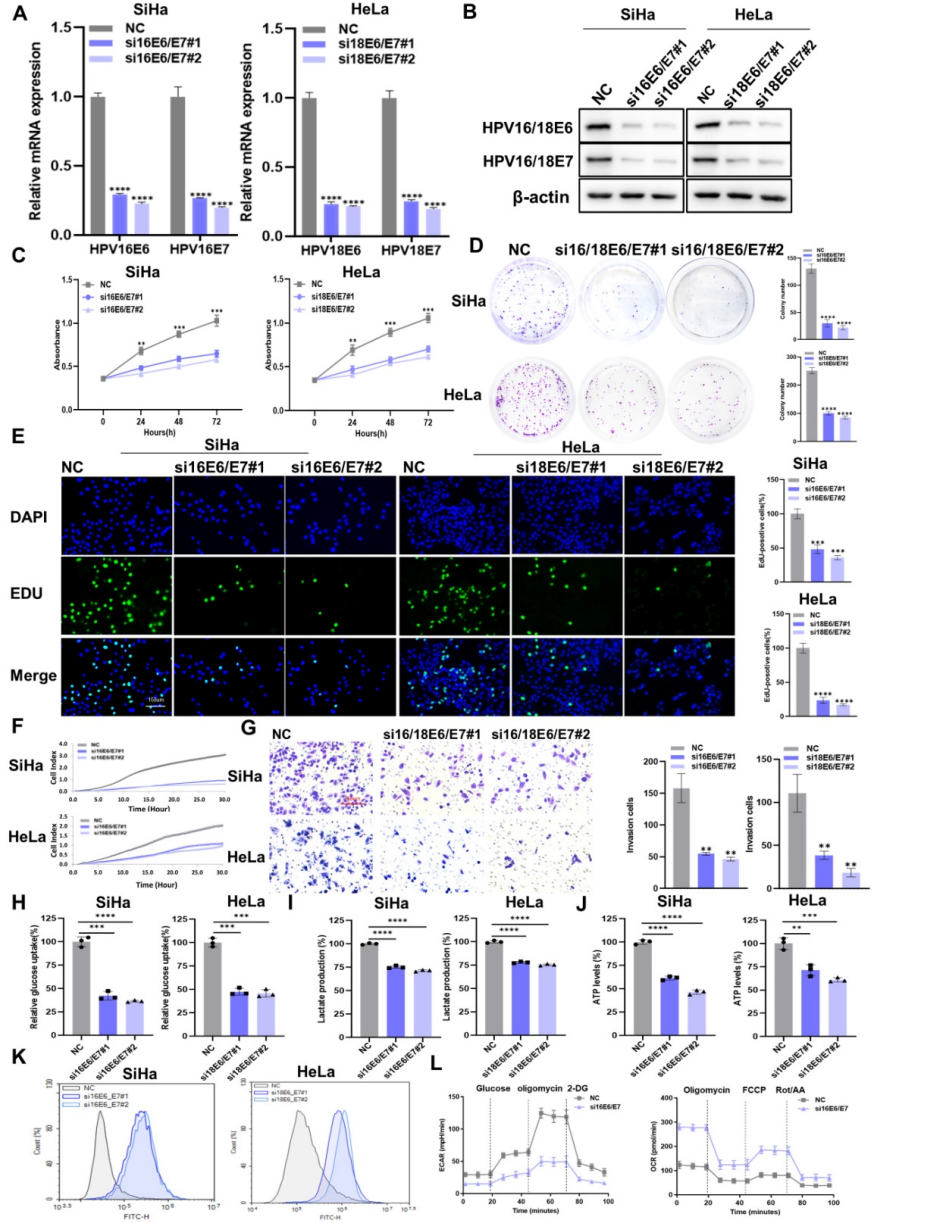
** The downregulation of HPV16/18 E6/E7 suppresses the proliferative, metastatic, and glycolytic potential of CC cells. (A, B)** SiHa and HeLa cells were transfected with HPV16/18 E6/E7 siRNA, following which E6/E7 mRNA (A) and protein (B) levels were determined by qPCR and western blot, respectively. **(C-E)** After HPV16/18 E6/E7 silencing, the viability (C), colony forming ability (D), and proliferative ability (5-ethynyl-2′-deoxyuridine [EdU] assay) (E) of the cells were evaluated. Scale bars: 150 µm. **(F, G)** The effects of HPV16/18 E6/E7 silencing on SiHa and HeLa cell migration and invasion (F) were monitored using a real-time cell analysis (RTCA) system and a transwell assay. Scale bars: 150 µm. **(H-K)** Glucose uptake (H), lactate production (I), ATP levels (J), and ROS content (K) in SiHa and HeLa cells were measured after HPV16/18 E6/E7 knockdown. **(L)** SiHa cells from (A) were used to detect the extracellular acidification rate (ECAR) and oxygen consumption rate (OCR) as indicators of glycolytic fluxes. Each value represents the mean ± SD of triplicate samples (Student's t-test). **P < 0.01, ***P < 0.001, and ****p < 0.0001.

To explore the effect of HPV16/18 E6/E7 on CC, we knocked down HPV16/18 E6/E7 in SiHa and Hela cells using siRNA. The transfection efficiency of HPV16/18 E6/E7 siRNA was measured by qRT-PCR (Figure 1A) and western blot (Figure 1B). HPV16/18 E6/E7 knockdown reduced the viability of CC cells (Figure 1C), as well as their colony-forming (Figure 1D) and proliferative (Figure 1E) abilities. The migratory and invasive ability of CC cells was detected using the RTCA system and transwell assays. The results showed that CC cells transfected with E6/E7 displayed reduced migratory (Figure 1F) and invasive (Figure 1G) potential compared with that in control cells. To investigate whether HPV16/18 E6/E7 could enhance aerobic glycolysis in CC, changes in the levels of glycolysis-related metabolites were assessed. As shown in Figure 1H-J, glucose uptake, lactate production, and ATP levels were decreased following E6/E7 knockdown. ROS is a byproduct of metabolism 26 that can impair normal cellular function and is closely linked with apoptosis and cell cycle arrest 27. Here, we found that intracellular ROS levels were higher in E6/E7-depleted SiHa and Hela cells than in control cells (Figure 1K). Flow cytometric analysis showed that the rates of cell apoptosis (Figure S2A) and cell cycle arrest (Figure S3A) were higher in E6/E7-knockdown cells than in the controls. Furthermore, HPV16 E6/E7 knockdown in SiHa cells was accompanied by a lower ECAR and a higher OCR (Figure 1L). The ECAR reflects aerobic glycolysis flux while the OCR is indicative of the state of mitochondrial oxidative respiration 28. Taken together, our findings showed that HPV16/18 E6/E7 enhances the proliferative, metastatic, and aerobic glycolytic capacity of CC cells.

### IGF2BP2 is a downstream target of HPV16/18 E6/E7 and is associated with tumor stage in CC patients

**Figure 2:**
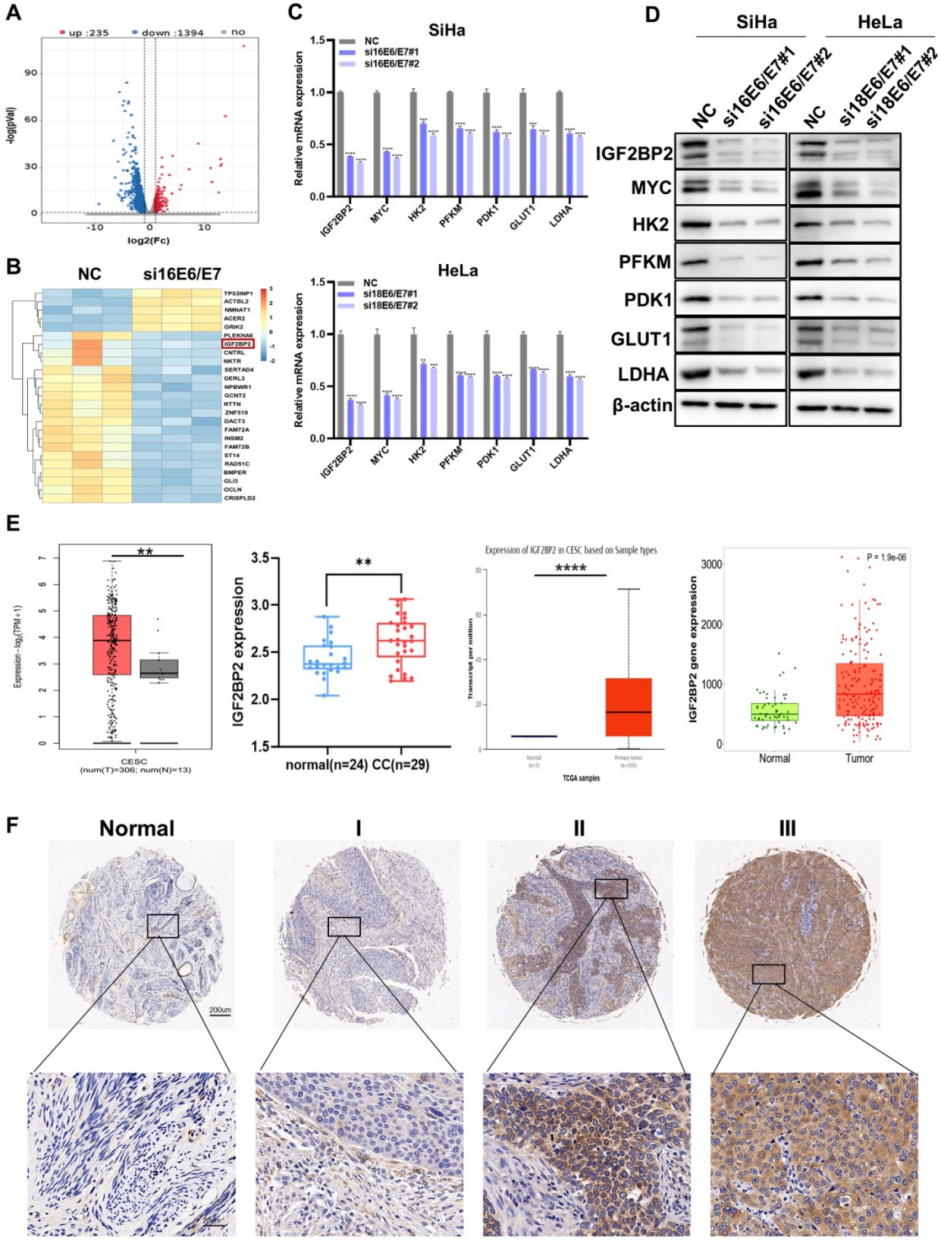
** IGF2BP2 is a downstream target of HPV16 E6/E7 and is associated with tumor stage in patients with CC. (A)** An enhanced volcano plot was generated using the OmicStudio tools at https://www.omicstudio.cn/tool. The red and blue dots represent upregulated and downregulated genes, respectively (fold change >2, *p* < 0.05, *n*=3). **(B)** A heat map was constructed based on the genes differentially expressed between the control and HPV16 E6/E7-knockdown cells. **(C, D)** qRT-PCR (C) and western blot (D) analysis of potential target genes and their products in SiHa and HeLa cells. **(E)** Differences in IGF2BP2 expression between CC tissue and normal tissue were analyzed by UALCAN (http://ualcan.path.uab.edu/), TNMplot (https://tnmplot.com/analysis/), GEPIA (cancer-pku.cn), and CESC microarrays (GSE63514). **(F)** The expression of IGF2BP2 in para-carcinoma tissues and CC tumor tissues at various clinical stages as determined by IHC analysis. Each value represents the mean ± SD for triplicate samples (Student *t* test). **P < 0.01, ***P < 0.001, and ****p < 0.0001.

To probe the molecular mechanisms involved in E6/E7-driven CC progression, RNA-seq was performed in HPV16 E6/E7-knockdown and control SiHa cells. Compared with that in NC cells, the expression levels of 1,394 genes were significantly downregulated following E6/E7 knockdown (Figure 2A). The heat map of the differential genes is shown in Figure 2B, identifying IGF2BP2 as a downstream target of HPV16 E6/E7. IGF2BP2 is a member of Insulin-like growth factor 2 mRNA-binding proteins (IGF2BPs) and belongs to an evolutionary conserved family of RNA-binding oncofetal proteins 29. GO analysis further showed significant enrichment of genes related to RNA binding (Figure S1A). Subsequently, the mRNA and protein levels were verified by qRT-PCR and western blot, respectively. The results showed that both the mRNA and proteins levels of IGF2BP2, MYC, and glycolysis-related genes were decreased in CC cells (Figure 2C and 2D). The expression of IGF2BP2 was further analyzed via various databases, including UALCAN, TNMplot, the GEPIA dataset, and CESC microarrays (GSE63514). We found that the expression of IGF2BP2 in CC tissue was significantly higher than that in matching normal tissue (Figure 2E). Similar results were obtained for IGF2BP2 IHC staining of the CC TMA (Figure 2F).

### IGF2BP2 is upregulated in CC and promotes MYC expression by recognizing the m^6^A-modified site in MYC RNA in CC cell lines

**Figure 3:**
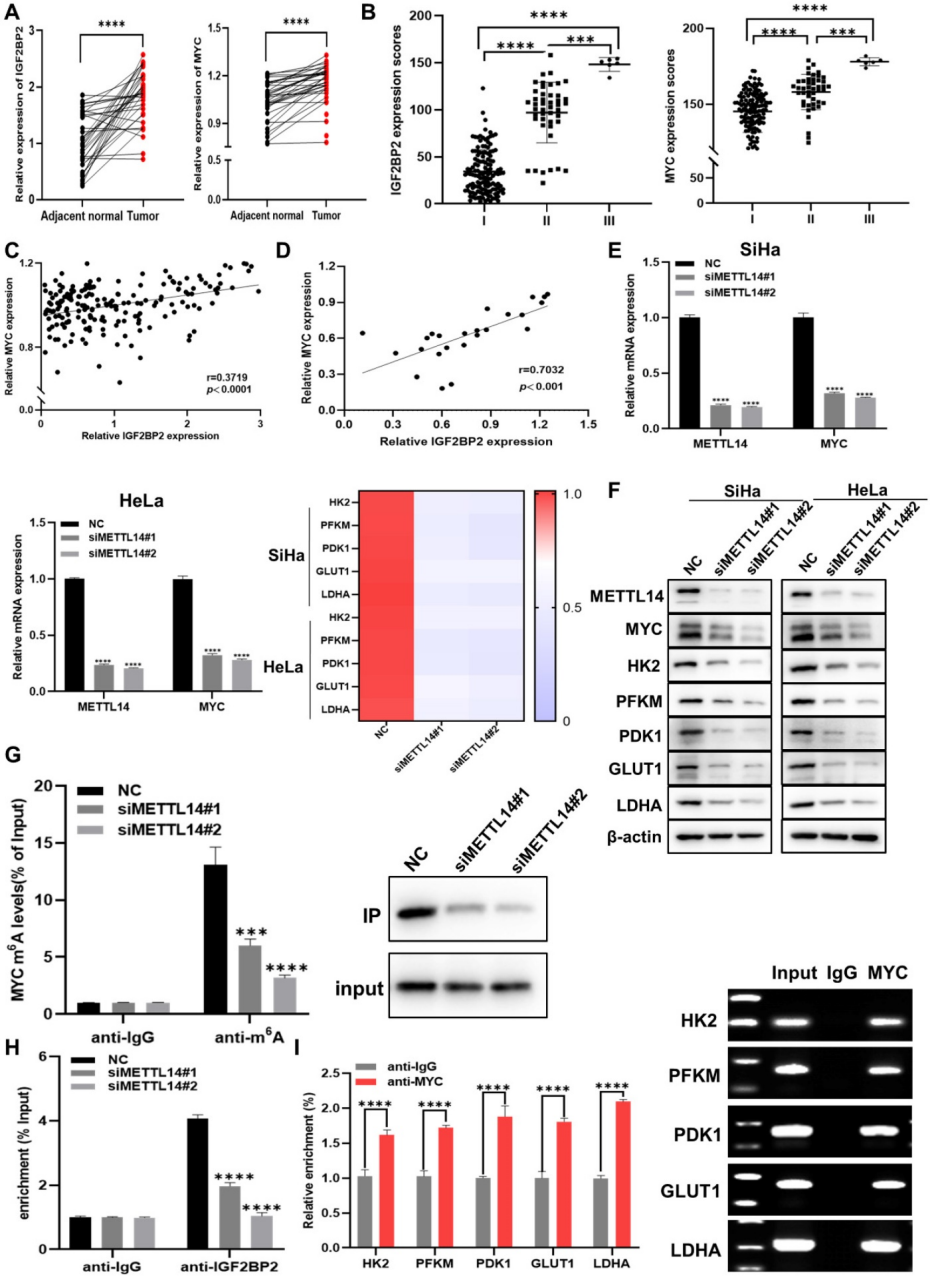
** IGF2BP2 is upregulated in CC and promotes MYC expression by recognizing m^6^A modifications in MYC mRNA in CC cells. (A)** Detection of IGF2BP2 and MYC expression in human CC tissues and adjacent normal tissues (*n*=38). **(B)** The expression of IGF2BP2 and MYC in 186 CC tumor tissue samples. **(C, D)** Correlation between IGF2BP2 and MYC expression in CC in tissue microarrays and tissue specimens. **(E, F)** The relative mRNA levels of METTL14, MYC, and glycolysis-related genes were determined using qRT-PCR (E) and western blot (F). **(G)** Me-RIP and IP assays were used to detect the m^6^A level in MYC mRNA and m^6^A expression in SiHa cells after METTL14 silencing. **(H)** The binding of IGF2BP2 to MYC was verified by RIP assay. **(I)** A ChIP assay was performed in SiHa cells using IgG and anti-MYC antibody. Each value represents the mean ± SD for triplicate samples (Student *t* test). ***P < 0.001, and ****p < 0.0001.

Further analysis of the results of the CC TMA staining showed that, in the FDU961 array, IGF2BP2 and MYC staining was markedly stronger in CC tissue than in adjacent normal tissues (Figure 3A and Figure S1B). To determine whether clinical stage was associated with IGF2BP2 and MYC, the FDU1921 array, which has different CC features compared with FDU961, was then used for IHC. We found that IGF2BP2 and MYC expression was positively correlated with CC stage (Figure 2B, Table 1, and Table 2). Based on the samples of 186 CC patients in the CC TMA used in Figure 3B, the correlation between the H-scores of IGF2BP2 and MYC was analyzed. The scatter plot shows the correlation between IGF2BP2 and MYC, as shown in Figure 3C. Then, the protein levels of IGF2BP2 and MYC in 24 CC samples were measured by western blotting, and the expression were quantified by ImageJ software (Figure 3D and Figure S1C). METTL14 is a non-catalytic subunit of the N6-adenosine-methyltransferase complex known to promote tumor progression by regulating mRNA function and stabilizing mRNA transcripts 30, 31. The mRNA (Figure 3E) and protein levels (Figure 3F) of MYC and glycolysis-related factors were significantly decreased when METTL14 was knocked down in CC cells. To determine whether MYC expression was affected by reduced m^6^A levels, we performed m^6^A-RIP in METTL14-knockout SiHa cells. qRT-PCR and IP assays showed that m^6^A-modified MYC expression and the m^6^A level were significantly downregulated in these cells (Figure 3G). The RIP results further showed that the levels of IGF2BP2-bound m^6^A-MYC mRNA were decreased in METTL14-silenced SiHa cells (Figure 3H), suggesting that IGF2BP2 regulates MYC in a m^6^A modification-dependent manner.

MYC is a key regulator of aerobic glycolysis, and its abnormal expression can impair cell metabolism 32. Using Cistrome Data Browser to analyze ChIP-seq data, it was found that aerobic glycolysis pathway genes were regulated by MYC. The ChIP-qPCR showed that MYC could directly bind to the promoter regions of HK2, PFKM, PDK1, GLUT1, and LDHA (Figure 3I). Taken together, these findings suggested that IGF2BP2 regulates the aerobic glycolysis pathway in CC cells by recognizing MYC m^6^A modification sites.

### IGF2BP2 silencing attenuates CC cell proliferation, migration, invasion, and glycolytic capacity

**Figure 4:**
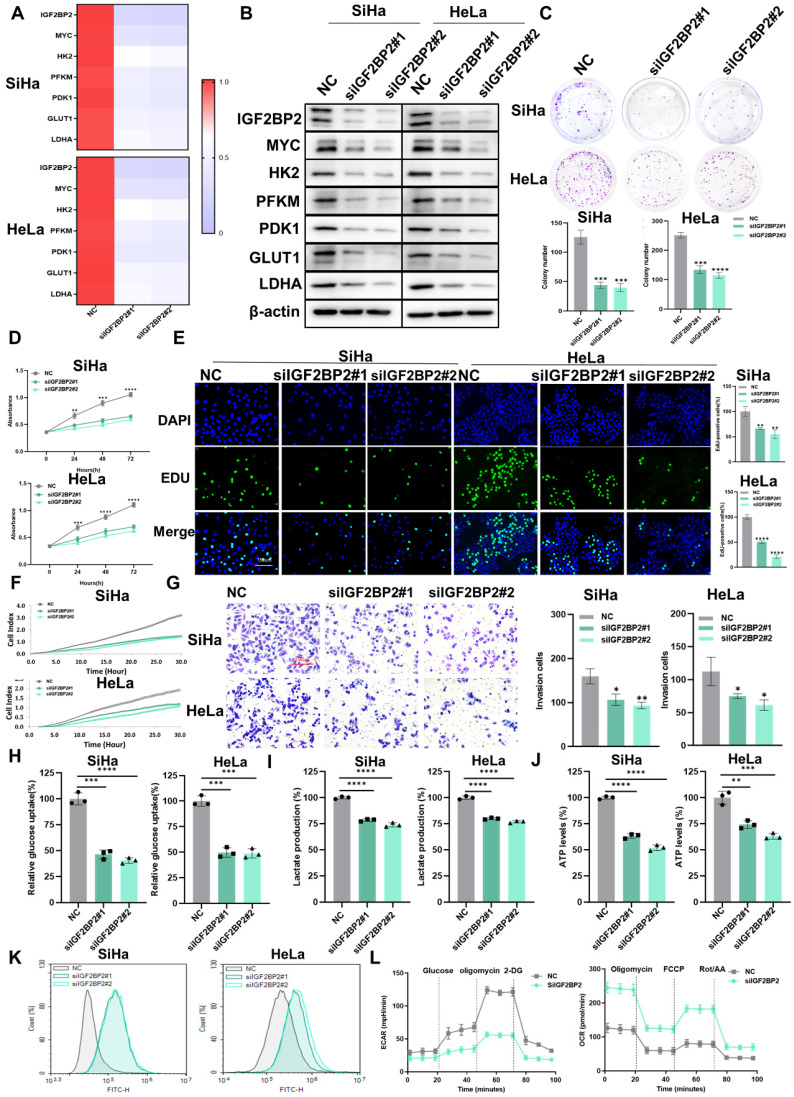
** IGF2BP2 silencing attenuated CC cell proliferation, migration, invasion, and glycolytic capacity. (A, B)** SiHa and HeLa cells were transfected with IGF2BP2-targeting siRNAs, following which mRNA and protein levels were determined by qPCR (A) and western blot (B), respectively. **(C-G)** The effects of IGF2BP2 silencing on the clonogenicity (C), growth (D), and proliferative (E), migratory (F), and invasive (G) abilities of SiHa and HeLa cells were evaluated. **(H-K)** SiHa and HeLa cells were transfected with siIGF2BP2, after which glucose uptake (H), lactate production (I), ATP levels (J), and intracellular ROS content (K) were determined. **(L)** The effect of knocking down IGF2BP2 on the extracellular acidification rate (ECAR) and oxygen consumption rate (OCR) in SiHa cells. Each value represents the mean ± SD of triplicate samples (Student's *t* test). *P < 0.05, **P < 0.01, ***P < 0.001, and ****p < 0.0001.

Next, we explored whether IGF2BP2 is involved in CC progression and aerobic glycolysis. qRT-PCR (Figure 4A) and western blotting (Figure 4B) results confirmed the expression of IGF2BP2 and the corresponding downstream molecules in IGF2BP2-knockdown cells was reduced. Compared with the control group, the clonogenicity (Figure 4C) and the capacity for growth (Figure 4D), proliferation (Figure 4E), migration (Figure 4F), and invasion (Figure 4G) of SiHa and HeLa cells was decreased with IGF2BP2 silencing. Moreover, the rates of cell apoptosis were increased in IGF2BP2-knockdown CC cells (Figure S2B), while the cell cycle was arrested at the G0/G1 phase (Figure S3B). That IGF2BP2 exerts a regulatory effect on MYC expression suggests that IGF2BP2 can affect intracellular glycolytic metabolism through glycolytic enzymes. To test this, glucose uptake, lactate production, ATP levels, and ROS content were measured in IGF2BP2-depleted SiHa and HeLa cells. Compared with the controls, IGF2BP2 knockdown in CC cells reduced glucose uptake (Figure 4H), lactate production (Figure 4I), and ATP levels (Figure 4J), but increased intracellular ROS content (Figure 4K). Consistent with these observations, the ECAR was decreased, whereas the OCR was increased, in IGF2BP2-knockdown SiHa cells compared with that in the NC group (Figure 4L). These results suggested that IGF2BP2 promotes aerobic glycolysis in CC cells and enhances their proliferative and metastatic potential.

### The overexpression of IGF2BP2 partially rescued the HPV16/18 E6/E7 knockdown-mediated attenuation of CC cell proliferation, metastasis, and aerobic glycolysis

**Figure 5:**
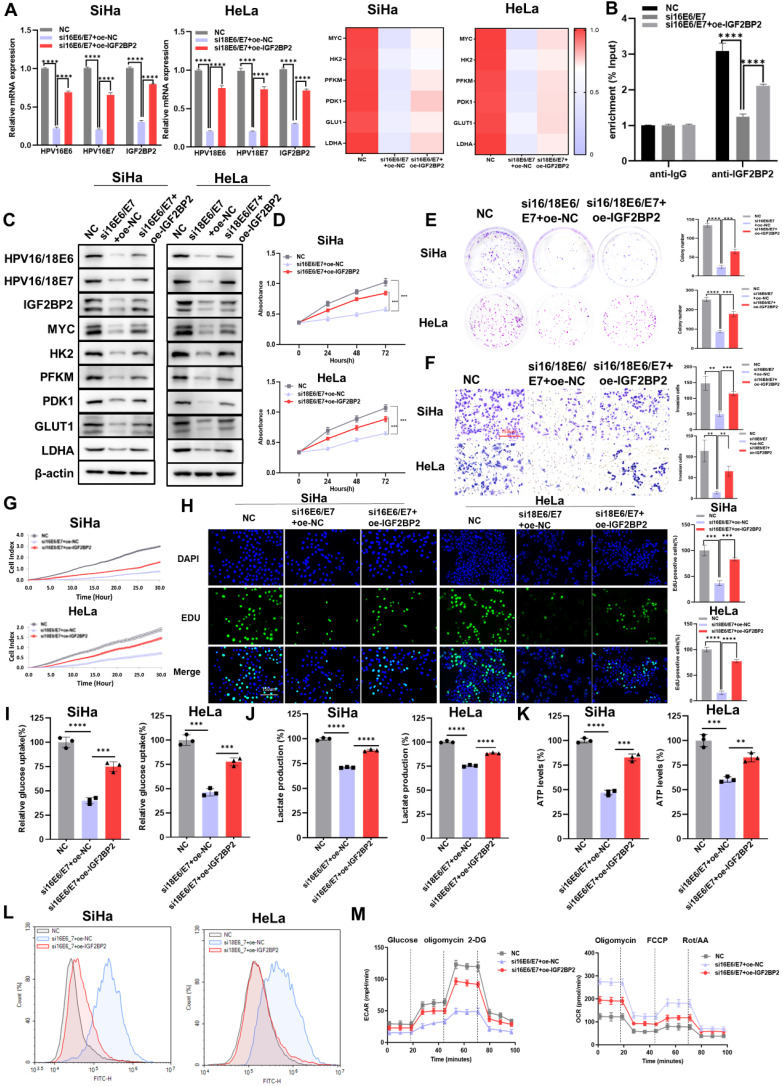
** The overexpression of IGF2BP2 partially rescued the HPV16/18 E6/E7 knockdown-mediated reduction in CC cell proliferation, metastasis, and aerobic glycolysis. (A)** qRT-PCR-based evaluation of the mRNA levels of IGF2BP2 and those of downstream factors following the transfection of an IGF2BP2 expression plasmid. **(B)** The relationship between HPV16 E6/E7 and MYC was assessed using a RIP assay. **(C)** SiHa and HeLa cells expressing siHPV16/18 E6/E7 were transfected with IGF2BP2, after which the protein levels were determined using western blot. **(D, E)** Cell viability and clonogenicity were determined in siHPV16/18 E6/E7-expressing SiHa and HeLa cells transfected or not with oe-IGF2BP2 were assessed by CCK-8 assay (D) and colony formation assay, respectively (E). **(F, G)** Migration (F) and invasion assays (G) of siHPV16/18 E6/E7-expressing SiHa and HeLa cells transfected with oe-IGF2BP2 or oe-NC. **(H)** Cell proliferation was monitored using an EdU staining assay. **(I-L)** IGF2BP2 was overexpressed in SiHa and HeLa cells transfected with siHPV16/18 E6/E7, following which glucose uptake (I), lactate production (J), ATP levels (K), and intracellular ROS content (L) were determined. **(M)** The ECAR and OCR were detected in CC cells expressing NC, siHPV16/18 E6/E7+oe-NC, or siHPV16/18 E6/E7+oe-IGF2BP2. Each value represents the mean ± SD for triplicate samples (Student *t* test). **P < 0.01, ***P < 0.001, and ****p < 0.0001.

As IGF2BP2 may be a downstream target of E6/E7, we investigated the effect of overexpressing IGF2BP2 on CC cells. First, we evaluated the transfection efficacy of the IGF2BP2 expression plasmid (oe-IGF2BP2) by qRT-PCR and western blot, and found that the decrease in the expression of E6/E7, IGF2BP2, and glycolysis-related genes was rescued in E6/E7-knockout cells transfected with oe-IGF2BP2 at both the mRNA (Figure 5A) and protein levels (Figure 5C). The results of the RIP assay showed that although the levels of MYC mRNA/IGF2BP2 binding were reduced in HPV16 E6/E7-silenced cells, this effect was rescued when these cells were transfected with oe-IGF2BP2 (Figure 5B). Next, CCK-8 and colony formation assays were performed to determine whether overexpressing IGF2BP2 could rescue the loss of CC cell viability and clonogenicity resulting from the knockdown of E6/E7 *in vitro*. We found that both the viability (Figure 5D) and colony-forming ability (Figure 5E) of the cells were rescued when IGF2BP2 was overexpressed. To assess the effect of IGF2BP2 on the metastatic and proliferative potential of shHPV16/18 E6/E7-expressing CC cells, transwell, EdU, and RTCA were performed.

The results showed that overexpressing IGF2BP2 could rescue the HPV16/18 E6/E7-knockdown-induced reduction in the invasive (Figure 5F), migratory (Figure 5G), and proliferative (Figure 5H) ability of CC cells. Similar results were obtained following the flow cytometric analysis of apoptosis (Figure S2C) and the cell cycle (Figure S3C). To determine if the progression of CC involves dysregulated glycolysis, we measured the production of major metabolites in the glycolytic pathway. As expected, the overexpression of IGF2BP2 rescued glucose uptake (Figure 5I), lactate production (Figure 5J), and ATP levels (Figure 5K) in E6/E7-knockdown CC cells, while reducing the intracellular ROS content (Figure 5L). Compared with siE6/E7-expressing SiHa cells, the ECAR of siE6/E7+oe-IGF2BP2-expressing SiHa cells was increased, almost reaching the levels observed in the NC-expressing cells; however, the OCR was decreased (Figure 5M). Combined, these results indicated that IGF2BP2 could reverse the effects of HPV16/18 E6/E7 knockdown in CC cells (loss of proliferative, metastatic, and aerobic glycolytic potential), at least to some extent.

### HPV 16 E6/E7 and IGF2BP2 enhance CC tumor growth *in vivo*

**Figure 6:**
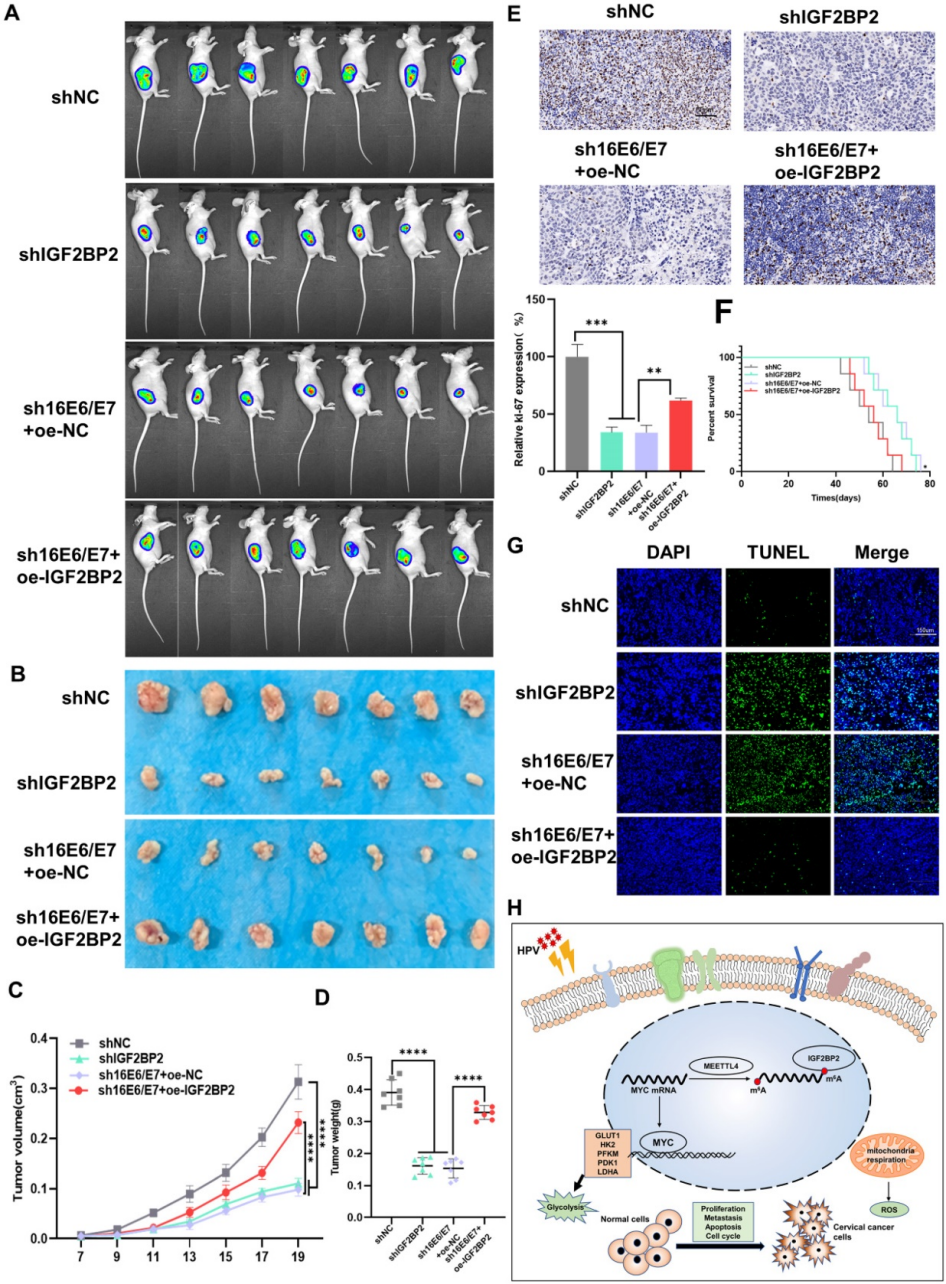
** HPV16 E6/E7 and IGF2BP2 enhance CC tumor growth *in vivo*. (A, B)** SiHa cells stably expressing shHPV16 E6/E7, shIGF2BP2, shHPV16 E6/E7+oe-IGF2BP2, or shNC were subcutaneously injected into the right flank of nude mice. Tumor images were acquired using the PerkinElmer IVIS preclinical *in vivo* imaging system 20 days (A) and 30 days (B) after injection. **(C)** From day 7 after injection, the tumor volume was measured every two days. **(D)** The weight of the xenografted tumors was measured. **(E)** Tumor sections underwent IHC staining using antibodies against Ki-67. Scale bars: 50 µm. **(F)** Kaplan-Meier survival curve showing the overall survival rate of mice in each group (*n*=7, *p < 0.05 by log-rank test). **(G)** Representative image of TUNEL staining, green indicates TUNEL-positive cells. Scale bars: 150 µm. **(H)** A proposed regulatory model of the role of IGF2BP2 in glycolysis and tumorigenesis. Each value represents the mean ± SD (Student's *t* test). *p < 0.05, **p < 0.01, ***p < 0.001, and ****p < 0.0001.

To investigate the role of E6/E7 and IGF2BP2 *in vivo*, SiHa cells expressing shIGF2BP2, shHPV16 E6/E7+oe-NC, shHPV16 E6/E7+oe-IGF2BP2, or shNC were subcutaneously injected into 5-week-old nude mice. One week after the injection, the mice were observed daily, and tumors were imaged *in vivo* on day 20. We found that the injection of SiHa cells expressing shIGF2BP2 or shHPV16 E6/E7+oe-NC reduced the tumor size to a similar degree, whereas tumor size was slightly increased in animals injected with shHPV16 E6/E7+oe-IGF2BP2-expressing SiHa cells (Figure 6A). Similar results were seen 30 days after injection (Figure 6B). Xenograft tumor volumes were measured every two days and a growth curve was plotted. Tumor growth was significantly inhibited in mice injected with shIGF2BP2, while IGF2BP2 overexpression, but not oe-NC, reversed the shHPV16 E6/E7-induced tumor growth attenuation (Figure 6C). At the end of the experiment, the mice were sacrificed and the tumors were removed. As shown in Figure 6D, shHPV16E6/E7+oe-NC and shIGF2BP2 attenuated tumor weight, whereas shHPV16 E6/E7+oe-IGF2BP2 increased the tumor weight. The results of IHC staining for Ki-67, used as a marker for evaluating tumor proliferation, were consistent with the above findings (Figure 6E). Kaplan-Meier survival curve analysis showed that survival time was increased in mice injected with shHPV16E6/E7+oe-NC- or shIGF2BP2-expressing SiHa cells, but was reduced in mice injected with shHPV16 E6/E7+oe-IGF2BP2 (Figure 6F). TUNEL staining further showed that the knockdown of HPV16 E6/E7 and IGF2BP2 promoted cell apoptosis, whereas IGF2BP2 overexpression elicited the opposite effect (Figure 6G). Figure 6H shows a proposed model of the role of IGF2BP2 in CC, whereby HPV E6/E7 protein regulates MYC m^6^A modification by targeting IGF2BP2, thus promoting aerobic glycolysis and tumor progression in CC.

## Discussion

Since the mid-20^th^ century, CC-associated incidence and mortality have declined significantly in the United States, mainly due to extensive screening practices and increased HPV vaccination rates 1. However, cervical screening programs are poorly implemented in 78 low- and middle-low-income countries 33. Because metastatic CC is generally not curable 34, early screening and identifying new molecular targets is of great significance for the prevention and treatment of this disease. In the present study, the potential roles of E6/E7 in CC progression were investigated both *in vitro* and *in vivo*, while the effect of E6/E7 on aerobic glycolysis was explored for the first time. Our results identified possible therapeutic strategies for CC treatment involving the targeting of metabolic pathways.

Radiotherapy is an effective method for the treatment of CC, but radiation resistance inevitably develops 35. HPV E6 and E7 antibodies are late markers of the disease, the levels of which increase with clinical stage progression 36. Because tumor-specific E6 and E7 can be recognized and attacked by the human adaptive immune system, they represent attractive therapeutic targets 34. The depletion of LKB1 was reported to promote the lung metastasis of HPV16 E6/E7- and K-RAS-transformed TC-1 cells and LKB1 countered HPV-induced metabolic reprogramming in CC through a common pathway involving c-MYC and HK2 37. Ma et al. found that E6/E7 expression and glycolytic activity were both increased in 5-FU-resistant CC cells, indicating that targeting the glycolytic pathway may be an effective strategy to attenuate resistance to chemotherapeutic drugs 38. In our study, the glycolytic, proliferative, and metastatic capacity of HPV16/18 E6/E7-knockdown CC cells was significantly decreased. RNA-seq analysis identified IGF2BP2 as a downstream target of HPV16/18 E6/E7, suggesting that HPV16/18 E6/E7 may affect CC progression by directly regulating IGF2BP2. Moreover, the results of CC tissue microarray IHC staining indicated that IGF2BP2 and MYC expression levels are higher in CC tissue than in adjacent tissue and are positively correlated with the clinical stage of CC.

IGF2BPs constitute a family of carcinoembryonic proteins that regulate cell function in cancer and exert carcinogenic effects as m^6^A readers 14, 30. In breast cancer, IGF2BP2 interacts with the antisense transcription pseudogene RPSAP52 to promote its binding to HMGA2 mRNA targets as well as protein translation through self-renewal 15. Ma et al reported that FGF13-AS1 inhibits stem cell characteristics and the glycolytic capacity of breast cancer cells through IGF2BPs 39. Moreover, circNDUFB2 is involved in the degradation of IGF2BPs and the activation of anti-tumor immunity in non-small cell lung cancer by regulating protein ubiquitination and cellular immune responses 29. Dysregulated IGF2BP2 expression leads to the accumulation of oncogenic molecules, such as MYC, thus supporting the malignant state of cancer cells 30. The knockdown of lncRNA LINRIS was reported to attenuate MYC-mediated aerobic glycolysis downstream of IGF2BP2 in colorectal cancer cells 40. Ye et al. showed that MALAT1 promotes thyroid cancer progression by binding to miR-204, thereby upregulating IGF2BP2, and, consequently, also MYC expression 41. In addition, the induction of lncMyoD can interfere with a positive feedback mechanism that exists between IGF2BP2 mRNA (such as IMP2, MYC, and NRAS) and IGF2BP2 protein, which provokes cell cycle arrest and promotes terminal cell differentiation 42. In the present study, the function of IGF2BP2 was investigated *in vitro* and *in vivo*. The results demonstrated that IGF2BP2 enhances the proliferative, metastatic, and aerobic glycolytic capacity of CC cells through binding to MYC m^6^A sites.

In cancer, active tumor cells use aerobic glycolysis as an energy source, even when mitochondrial function is intact 9. Studies in hepatocellular carcinoma have shown that enhanced cell growth and movement are driven by increased aerobic glycolysis induced by extracellular vesicles with reduced triosephosphate isomerase 1 43. Li et al. reported that the MYC-dependent downregulation of fructose-1,6-diphosphatase induced anticancer effects by regulating signal transducer and activator of transcription 3 (STAT3) in epithelial ovarian cancer 44. Because MYC is a key regulator of glycolysis and also a downstream target of IGF2BP2 30, we evaluated the effect of HPV16/18 E6/E7 and IGF2BP2 interaction on glycolysis in CC. Following E6/E7 knockdown in CC cells, the expression levels of glycolytic enzymes downstream of MYC decreased, as did aerobic glycolysis flux and ATP production. Meanwhile, overexpressing IGF2BP2 rescued the inhibition of aerobic glycolysis and CC progression resulting from E6/E7 knockdown, suggesting that IGF2BP2, a target of E6/E7, plays a bridging role between E6/E7 and MYC in the regulation of aerobic glycolysis.

In summary, we demonstrated that E6/E7 can regulate the aerobic glycolysis of CC cells through IGF2BP2-mediated modulation of m^6^A-MYC mRNA *in vitro* and *in vivo*. Given that cell metabolism is a complex process involving the activity of numerous genes, we cannot exclude that E6/E7 may also target aerobic glycolysis by influencing the methylation status of mRNAs other than MYC in an IGF2BP2-dependent manner. Our finding that the HPV E6/E7/IGF2BP2/m^6^A-MYC/glycolysis axis may play a key role in the pathogenesis of CC suggests that targeting HPV E6/E7 and downstream pathways may represent an attractive therapeutic option for the treatment of patients with this cancer.

## Supplementary Material

Supplementary figures and tables.

## Figures and Tables

**Table 1:** Correlation between IGF2BP2 expression and clinicopathological parameters in cervical cancer

	Low IGF2BP2		High IGF2BP2		*P*-value
	No.	%	No.	%	
All patients	93	50.00	93	50.00	
**Age (years)**					
≤40	21	11.29	23	13.98	
>40	72	38.71	70	36.02	0.73
**Lymph node**					
N0	93	50.00	89	47.85	
N1	0	0.00	4	2.15	0.121
**Grade**					
-	4	3.23	4	1.08	
1 or 2	59	26.34	56	35.48	
3 or 4	30	20.43	33	13.44	0.6382
**Stage**					
I	86	46.24	49	26.34	
II or III	7	3.76	44	23.66	**<0.0001**

No. indicates the number of cases. Values in bold indicate statistically significant differences.

**Table 2:** Correlation between MYC expression and clinicopathological parameters in cervical cancer

Characteristics	low MYC		high MYC		
	No.	%	No.	%	
All patients	93	50.00	93	50.00	
**Age (years)**					
≤40	21	11.29	26	13.98	
>40	72	38.71	67	36.02	0.73
**Lymph node**					
N0	93	50.00	89	47.85	
N1	0	0.00	4	2.15	0.121
**Grade**					
-	6	3.23	2	1.08	
1 or 2	49	26.34	66	35.48	
3 or 4	38	20.43	25	13.44	**0.0238**
**Stage**					
I	83	44.62	52	27.96	
II or III	10	5.38	41	22.04	**<0.0001**

No. indicates the number of cases. Values in bold indicate statistically significant differences.
