# Supplementary material for: HPV E6/E7 promotes aerobic glycolysis in cervical cancer by regulating IGF2BP2 to stabilize m6A-MYC expression
Source: Int J Biol Sci. 2022 Jan 1;18(2):507–21. doi: 10.7150/ijbs.67770 (PMC8741847; doi:10.7150/ijbs.67770)
Supplement: Supplementary file 1 — Supplementary figures and tables. [file ijbsv18p0507s1.pdf]

Supplementary Figure

Figure S1. (A) Statistics for GO enrichment analysis. (B) The expression of IGF2BP2 and MYC in human CC tissues and adjacent normal tissues (*n*=186). (C) Detection of IGF2BP2 and MYC protein expression in human CC tissues (*n*=24).

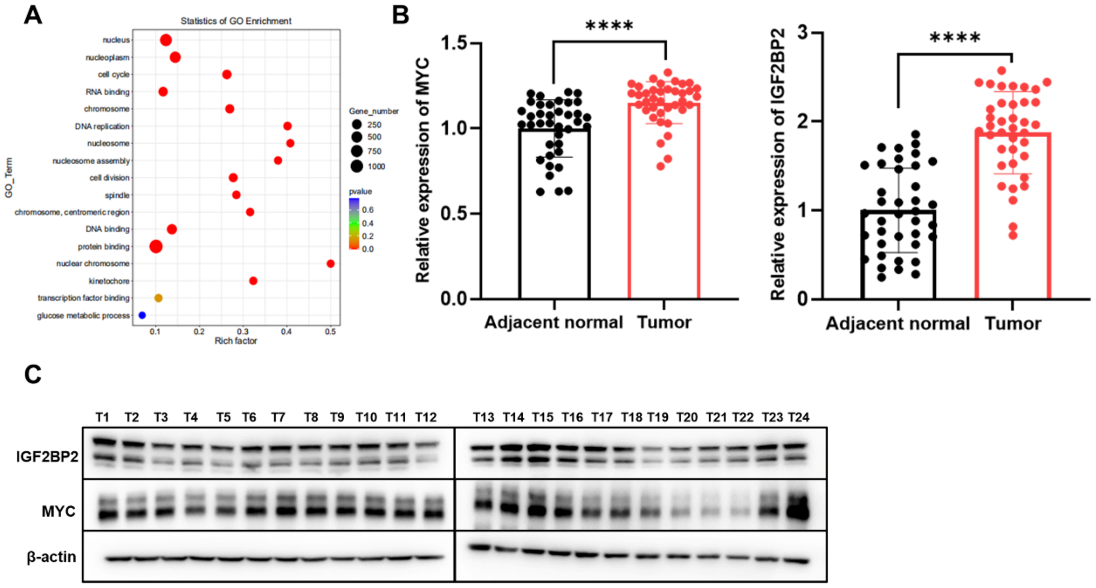

A
Statistics of GO Enrichment
nucleus
nucleoplasm
cell cycle
RNA binding
chromosome
DNA replication
nucleosome
nucleosome assembly
cell division
spindle
chromosome, centromeric region
DNA binding
protein binding
nuclear chromosome
kinetochore
transcription factor binding
glucose metabolic process
GO_Term
Rich factor
Gene_number
250
500
750
1000
pvalue
0.6
0.4
0.2
0.0
B
Relative expression of MYC
****
Adjacent normal
Tumor
Relative expression of IGF2BP2
****
Adjacent normal
Tumor
C
T1 T2 T3 T4 T5 T6 T7 T8 T9 T10 T11 T12
T13 T14 T15 T16 T17 T18 T19 T20 T21 T22 T23 T24
IGF2BP2
MYC
β-actin

Figure S2. The effect of HPV16/18 E6/E7 and IGF2BP2 on cell apoptosis. (A) Flow cytometry was used to detect cell apoptosis in SiHa and HeLa cells transfected with HPV16/18 E6/E7-targeting siRNA. (B) Flow cytometry was used to detect cell apoptosis in SiHa and HeLa cells transfected with IGF2BP2 siRNA. (C) Flow cytometry was used to detect cell apoptosis in HeLa cells transfected with different siRNAs and plasmids.

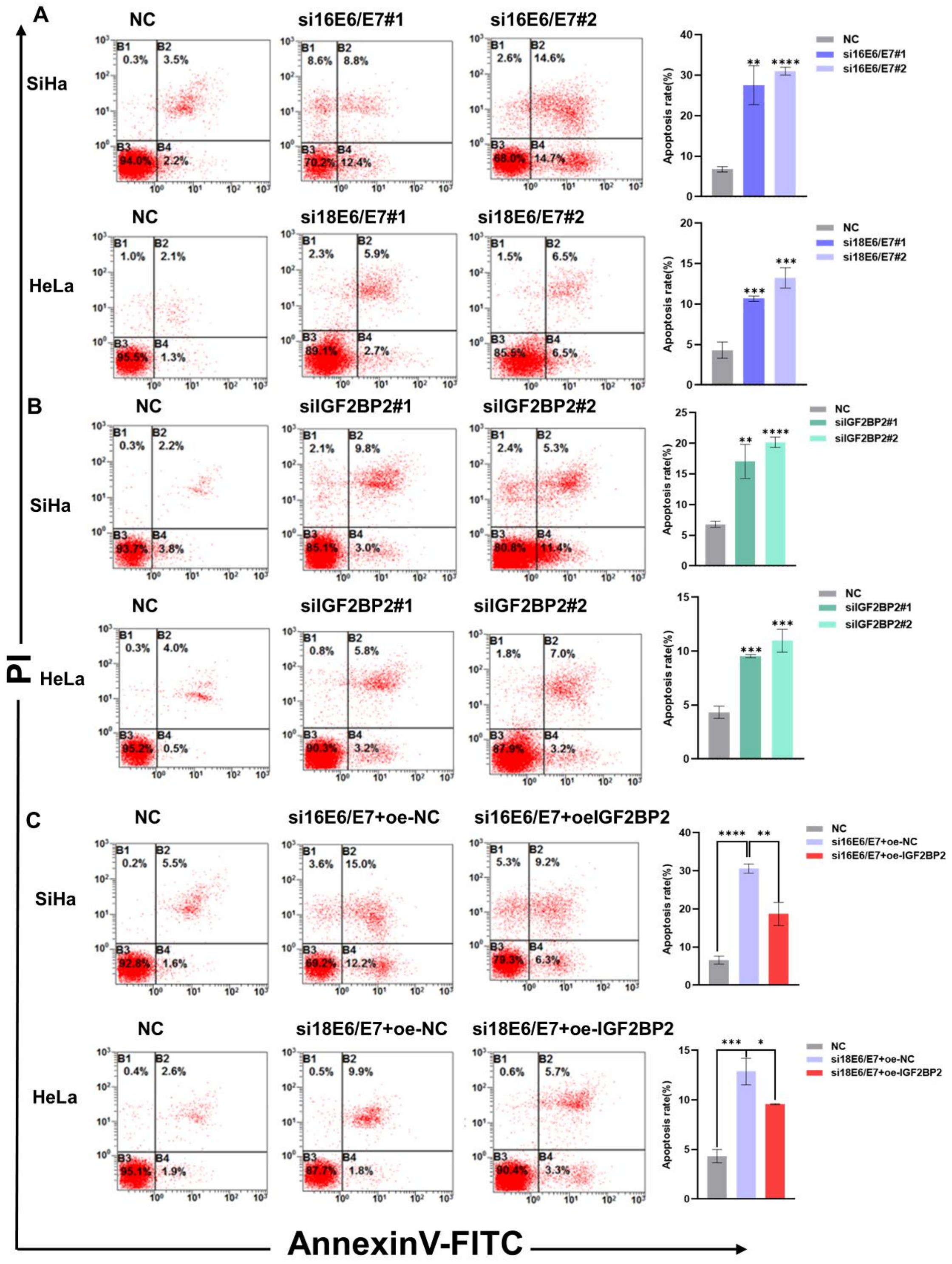

A
B
C
SiHa
HeLa
NC
si16E6/E7#1
si16E6/E7#2
si18E6/E7#1
si18E6/E7#2
siIGF2BP2#1
siIGF2BP2#2
si16E6/E7+oe-NC
si16E6/E7+oeIGF2BP2
si18E6/E7+oe-NC
si18E6/E7+oe-IGF2BP2
si16E6/E7+oe-IGF2BP2
si18E6/E7+oe-IGF2BP2
Apoptosis rate(%)
PI
AnnexinV-FITC

Figure S3. The effect of HPV16/18 E6/E7 and IGF2BP2 on the cell cycle. (A) The cell cycle in SiHa and HeLa cells transfected with HPV16/18 E6/E7-targeting siRNA was evaluated by propidium iodide (PI) staining using flow cytometry. (B) Flow cytometry was used to detect the cell cycle in SiHa and HeLa cells transfected with IGF2BP2 siRNA. (B) The cell cycle in SiHa and HeLa cells transfected with different siRNAs and plasmids was examined by PI staining using flow cytometry.

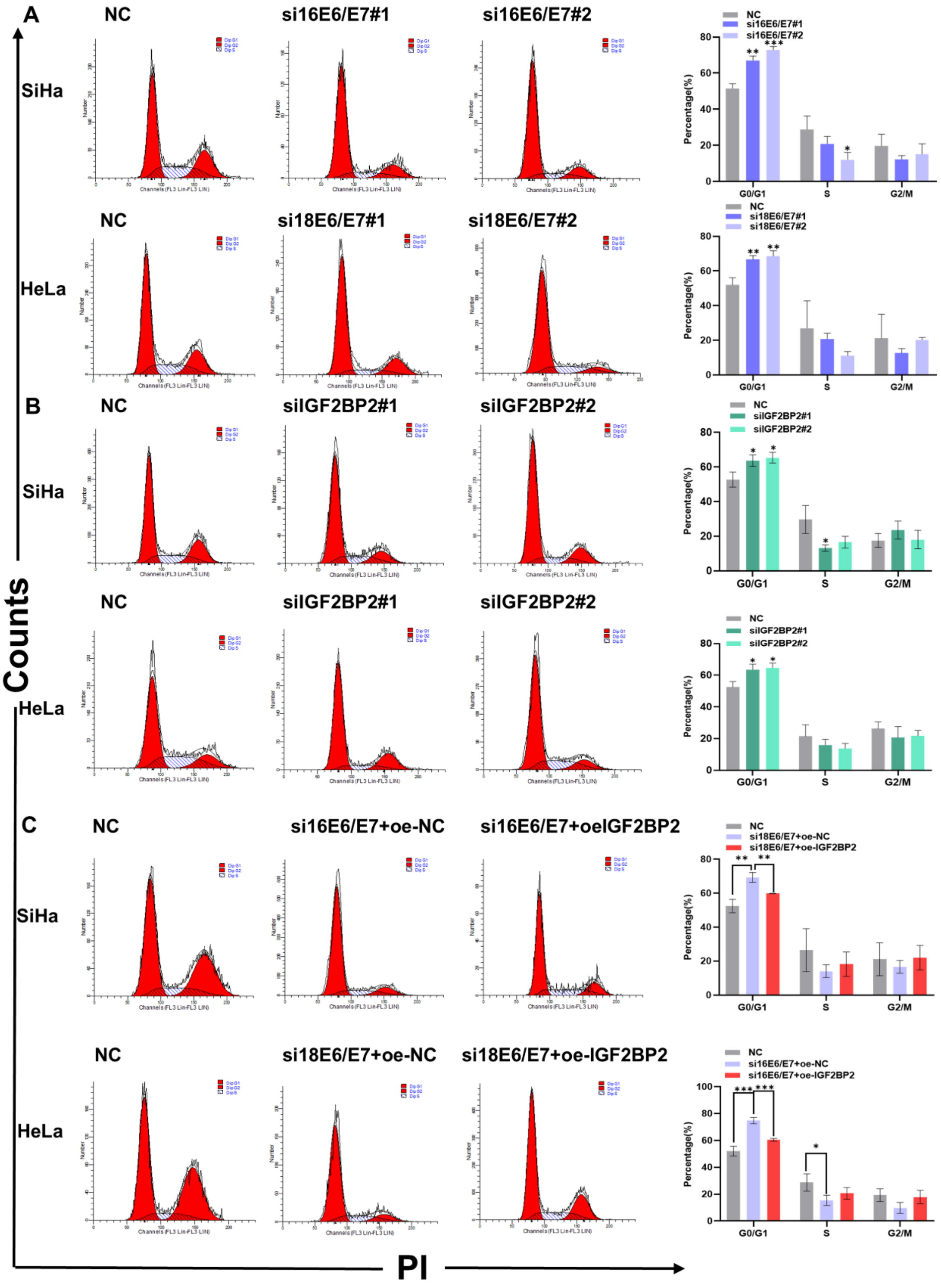

A
B
C
NC
si16E6/E7#1
si16E6/E7#2
si18E6/E7#1
si18E6/E7#2
siIGF2BP2#1
siIGF2BP2#2
si16E6/E7+oe-NC
si16E6/E7+oeIGF2BP2
si18E6/E7+oe-NC
si18E6/E7+oe-IGF2BP2
SiHa
HeLa
Counts
PI
Percentage(%)
G0/G1
S
G2/M
si18E6/E7+oe-IGF2BP2
si16E6/E7+oe-NC
si16E6/E7+oe-IGF2BP2

Supplementary Material

Supplementary Table 1 The sequences of siRNAs, shRNAs and plasmids

Plasmids	sequences (5' > 3')
si/shHPV16E6/E7#1	CCGGACAGAGCCCAUUACATT
siHPV16E6/E7#2	CAACUGAUCUCUACUGUUATT
si/shHPV18E6/E7#1	CCACAACGUCACACAAUGUTT
siHPV18E6/E7#2	UCCAGCAGCUGUUUCUGAATT
si/shIGF2BP2#1	AGTGAAGCTGGAAGCGCATAT
siIGF2BP2#2	TTCCCGCATCATCACTCTTAT
siMETTL14#1	GCTGGACTTGGGATGATATTA
siMETTL14#2	GAACCTGAAATTGGCAATATA
siNC/NC	UUCUCCGAACGUGUCACGUTT
pcDNA3.1(+)/IGF2BP2	ATGATGAACAAGCTTTACATCGGGAACCTGAGCCCCGCCGTCACCGCCGACGA<br>CCTCCGGCAGCTCTTTGGGGACAGGAAGCTGCCCCTGGCGGGACAGGTCCT<br>GCTGAAGTCCGGCTACGCCTTCGTGGACTACCCCGACCAGAACTGGGCCATC<br>CGCGCCATCGAGACCCTCTCGGGTAAAGTGGAATTGCATGGGAAAATCATGGA<br>AGTTGATTACTCAGTCTCTAAAAAGCTAAGGAGCAGGAAAATTCAGATTCGAAA<br>CATCCCTCCTCACCTGCAGTGGGAGGTGTTGGATGGACTTTTGGCTCAATATG<br>GGACAGTGGAGAATGTGGAACAAGTCAACACAGACACAGAAACCGCCGTTGT<br>CAACGTCACATATGCAACAAGAGAAGAAGCAAAAATAGCCATGGAGAAGCTAAG<br>CGGGCATCAGTTTGAGAACTACTCCTTCAAGATTTCCTACATCCCGGATGAAGA<br>GGTGAGCTCCCCTTCGCCCCCTCAGCGAGCCCAGCGTGGGGACCACTCTTCC<br>CGGGAGCAAGGCCACGCCCCTGGGGGCACTTCTCAGGCCAGACAGATTGATT<br>TCCCGCTGCGGATCCTGGTCCCCACCCAGTTTGTTGGTGCCATCATCGGAAAG<br>GAGGGCTTGACCATAAAGAACATCACTAAGCAGACCCAGTCCCGGGTAGATATC<br>CATAGAAAAGAGAACTCTGGAGCTGCAGAGAAGCCTGTCACCATCCATGCCAC<br>CCCAGAGGGGACTTCTGAAGCATGCCGCATGATTCTTGAAATCATGCAGAAAG<br>AGGCAGATGAGACCAAACTAGCCGAAGAGATTCCTCTGAAAATCTTGGCACAC<br>AATGGCTTGGTTGGAAGACTGATTGGAAAAGAAGGCAGAAATTTGAAGAAAATT<br>GAACATGAAACAGGGACCAAGATAACAATCTCATCTTTGCAGGATTTGAGCATAT<br>ACAACCCGGAAAGAACCATCACTGTGAAGGGCACAGTTGAGGCCTGTGCCAG<br>TGCTGAGATAGAGATTATGAAGAAGCTGCGTGAGGCCTTTGAAAATGATATGCT<br>GGCTGTTAACCAACAAGCCAATCTGATCCCAGGGTTGAACCTCAGCGCACTTG<br>GCATCTTTTCAACAGGACTGTCCGTGCTATCTCCACCAGCAGGGCCCCGCGGA<br>GCTCCCCCCGCTGCCCCCTACCA
	CCCCTTCACTACCCACTCCGGATACTTCTCCAGCCTGTACCCCCATCACCAGTT<br>TGGCCCGTTCCCGCATCATCACTCTTATCCAGAGCAGGAGATTGTGAATCTCTT<br>CATCCCAACCCAGGCTGTGGGCGCCATCATCGGGAAGAAGGGGGCACACATC<br>AAACAGCTGGCGAGATTCGCCGGAGCCTCTATCAAGATTGCCCCTGCGGAAGG<br>CCCAGACGTCAGCGAAAGGATGGTCATCATCACCGGGCCACCGGAAGCCCAG<br>TTCAAGGCCCAGGGACGGATCTTTGGGAAACTGAAAGAGGAAAACTTCTTTAA<br>CCCCAAAGAAGAAGTGAAGCTGGAAGCGCATATCAGAGTGCCCTCTTCCACAG<br>CTGGCCGGGTGATTGGCAAAGGTGGCAAGACCGTGAACGAACTGCAGAACTT

	AACCAGTGCAGAAGTCATCGTGCCTCGTGACCAAACGCCAGATGAAAATGAGG<br>AAGTGATCGTCAGAATTATCGGGCACTTCTTTGCTAGCCAGACTGCACAGCGCA<br>AGATCAGGGAAATTGTACAACAGGTGAAGCAGCAGGAGCAGAAATACCCTCAG<br>GGAGTCGCCTCACAGCGCAGCAAGTGA

Supplementary Table 2 Primer sequences

Genes	Forward primer (5′→3′)	Reverse primer (5′→3′)
MYC	CATCAGCACAACTACGCAGC	GCTGGTGCATTTTCGGTTGT
HK2	GTGAATCGGAGAGGTCCCAC	GCTAACTTCGGCCACAGGAT
LDHA	ACGTGCATTCCCGATTCCTT	AACAGCACCAACCCCAACAA
PFKM	AATCTGCAAGAAAGCAGCGG	TACCAACTCGAACCACAGCC
PDK1	CCAGGGCTGCTTGAGTGTTA	AGCCATTTACTGCCTGCCAT
SLC2A1	GGCTTCTCCAACTGGACCTC	CCGGAAGCGATCTCATCGAA
IGF2BP2	AGTGGAATTGCATGGGAAAATCA	CAACGGCGGTTTCTGTGTC
METTL14	AGTGCCGACAGCATTGGTG	GGAGCAGAGGTATCATAGGAAGC
HPV16 E7	CCGGACAGAGCCCATTACAA	TTTGTACGCACAACCGAAGC
HPV18 E6	GGGCACTATAGAGGCCAGTG	GTGTTTCTCTGCGTCGTTGG
HPV18 E7	AACATTTACCAGCCCGACGA	AGGTCGTCTGCTGAGCTTTC
HPV16 E6	CAGGAGCGACCCAGAAAGTT	GCAGTAACTGTTGCTTGCAGT
m^6A-MYC	GCATACATCCTGTCCGTCCA	GTCGTTTCCGCAACAAGTCC
β-actin	CTCCATCCTGGCCTCGCTGT	GCTGTCACCTTCACCGTTCC
GAPDH	AATCCCATCACCATCTTCCAG	AAATGAGCCCCAGCCTTC
CHIP-LDHA	GGAGGGCAGCACCTTACTTAG	TCTGGAAAGCGGCTCCTACA
CHIP-HK2	CCGCAGGTAGTCAGGGATTG	GAAGGAGAAGGGAACCGCTC
CHIP-PFKM	TACCCTGGCACTCACCCAAT	ACTCTTGGAGCTCATGACGG
CHIP-SLC2A1	GGCTCCTGACTCCTCCGC	AGACTCCTGCCCGCCTTAC
CHIP-PDK1	TGGCTGGCGGCGTAAATAA	ACTTCACAGCATCTTGTCCTCAT

Supplementary Table 3. Primary and secondary antibodies and dilution ratio

Target	Company	Cat.No.	Dilution ratio
HPV 16 E7	biorbyt	orb635192	1:500
HPV 18 E7	abcam	ab100953	1:500
HPV 16E6+HPV18 E6	abcam	ab70	1:500
IGF2BP2	Proteintech,China	11601-1-AP	1:3000
METTL14	Proteintech,China	26158-1-AP	1:2000
c-MYC	Proteintech,China	10828-1-AP	1: 2,000
HK2	Proteintech,China	22029-1-AP	1: 5,000
PFKM	Proteintech,China	55028-1-AP	1: 1,000
PDK1	Proteintech,China	10026-1-AP	1: 1,000
GLUT1	Proteintech,China	21829-1-AP	1: 1,000
LDHA	Proteintech,China;	19987-1-AP	1: 5,000
β-actin	Proteintech,China	60008-1-Ig	1: 5,000
anti-mouse HRP secondary antibody	Immunoway,USA	RS0001	1:5000
anti-rabbit HRP secondary antibody	Immunoway,USA	RS0002	1:5000
